# Speech and mastication difficulties: Clear aligners versus conventional braces

**DOI:** 10.6026/973206300220385

**Published:** 2026-01-31

**Authors:** Manche Meghana, Akhilesh M, Zeeshan Mubashir, Kommu Pavan, Simran Suhani, Balaji Dhanraj Kendre, Rahul Tiwari

**Affiliations:** 1 Department of Orthodontics and Dentofacial Orthopedics, Mamata Dental College, Khammam, Telangana, India; 2 Department of Orthodontics and Dentofacial Orthopaedics, Hyderabad, Telangana, India; 3 Department of Orthodontics and Dentofacial Orthopaedics, Al Jouf Specialised Dental Center, Ministry of Health, Sakaka, Aljouf Province, Kingdom of Saudi Arabia; 4 Department of Orthodontics and Dentofacial Orthopedics, Tirumala Institute of Dental Sciences and Research Centre, Nizamabad, Telangana, India; 5 Department of Orthodontics, Vananchal Dental College, Garhwa, Jharkhand, India; 6 Department of Orthodontics & Dentofacial Orthopedics, Saraswati Dhanwantari Dental College & Hospital, Parbhani, Maharashtra, India; 7 Department of Dental Research Cell, Dr. D. Y. Patil Dental College & Hospital, Dr. D. Y. Patil Vidyapeeth (Deemed to be University), Pimpri, Pune, Maharashtra, India

**Keywords:** Orthodontic appliances, clear aligners, mastication disorders, speech articulation, patient reported outcomes

## Abstract

Orthodontic appliances influence patients' oral functions, especially speech and mastication. Therefore, it is of interest to compare speech and mastication difficulties among patients treated with clear aligners and conventional braces. One hundred participants were assessed through a validated questionnaire and performance tests. Clear aligners showed fewer articulation and chewing problems than fixed braces. The differences were statistically significant. Thus, we show clear aligners may provide improved comfort and oral function during orthodontic treatment.

## Background:

Orthodontic treatment corrects dental irregularities and improves oral function. Fixed appliances such as metal or ceramic braces are traditionally used for controlled tooth movement. Demand for aesthetic and comfortable alternatives has led to clear thermoplastic aligners. These removable systems are popular due to discreet appearance and easier hygiene maintenance [1, 2-3]. They also cause less treatment-related discomfort. However, functional effects of aligners remain under investigation. Fixed appliances can interfere with speech due to brackets and wires. Aligners may affect pronunciation of fricative and sibilant sounds [4]. Masticatory performance may be reduced by occlusal interference or discomfort [5]. Several studies compared comfort, hygiene and aesthetics between aligners and braces [6, 7]. Limited evidence exists on speech and mastication efficiency [8, 9- 10]. These functions are closely related to quality of life and treatment compliance. Therefore, it is of interest to compare speech and mastication difficulties between aligners and conventional braces.

## Materials and Methods:

## Study design and participants:

A cross-sectional comparative study was conducted on 100 orthodontic patients (aged 18-30 years) undergoing treatment for at least 3 months at a university dental hospital. Participants were divided equally into two groups:

[1] Group A: Clear aligner users (n = 50)

[2] Group B: Conventional fixed braces users (n = 50)

## Inclusion criteria:

[1] Class I or mild Class II malocclusion

[2] No craniofacial deformity or systemic disease affecting muscle function

[3] Normal hearing and native fluency in English/Hindi

## Data collection:

Speech and mastication difficulties were evaluated using:

[1] Speech assessment: A standardized speech intelligibility test with 50 phonetically balanced words scored by three blinded evaluators.

[2] Masticatory performance: A color-changeable chewing gum test quantifying color variation after 20 chewing strokes.

[3] Patient self-reported questionnaire: Visual analog scale (VAS, 0-10) assessing perceived difficulty in speech and chewing.

## Statistical analysis:

Descriptive data were expressed as mean ± SD. Intergroup comparisons were performed using independent t-tests and Chi-square tests. Significance was set at p < 0.05. Statistical analysis was carried out using SPSS v26.

## Results:

Patients wearing clear aligners demonstrated better speech intelligibility than those with fixed braces. They exhibited fewer articulation errors during phonetic assessment. The mean subjective speech difficulty score was lower in the aligner group. Detailed comparison of speech parameters is presented in Table 1. Aligner users showed significantly greater masticatory efficiency than brace users. They also reported less subjective chewing difficulty. Fixed appliances limited occlusal contact during function and increased discomfort during mastication. Comparative mastication outcomes are summarized in Table 2. Statistical analysis confirmed that clear aligners outperformed conventional braces in both speech clarity and chewing ability. No gender-based differences were observed. The aligner group adapted to articulation and mastication within two weeks, while the brace group required more than four weeks for comparable adaptation.

## Discussion:

The findings highlight that clear aligners minimize speech and masticatory difficulties compared to fixed appliances. These results align with earlier reports emphasizing patient comfort and functional ease with aligners [11, 12-13]. Speech articulation depends on coordinated tongue-palate interaction. Fixed braces alter palatal contour and lead to distortions in sibilant and alveolar consonants [10]. Clear aligners are thin and smooth and allow easier neuromuscular adaptation. This explains the higher speech intelligibility observed in the aligner group. Similar studies comparing aligners with other fixed appliances have reported fewer speech errors with aligners [11]. Mastication is influenced by occlusal contact and neuromuscular coordination. Fixed brackets restrict mandibular movements and create transient discomfort, impairing chewing efficiency [12]. Aligner users in the present study demonstrated higher masticatory performance. This finding supports evidence that removable appliances reduce occlusal interference and muscle fatigue [13]. Improved periodontal status and lower inflammatory response associated with aligners may further facilitate efficient chewing [14, 15-16]. Faster adaptation and lower self-reported difficulty scores indicate superior patient-centered outcomes. Aligner removability allows better oral hygiene and unrestricted food choice. This minimizes interference in social and professional communication [17, 18]. Understanding functional outcomes assists clinicians in selecting appliances based on patient needs. Aligners may be preferred for individuals prioritizing comfort and speech clarity. Fixed appliances remain necessary for complex malocclusions. Future longitudinal studies using electromyographic and acoustic analyses may provide deeper insight into long- term neuromuscular adaptation [19, 20].

## Conclusion:

Clear aligners cause fewer speech articulation and mastication difficulties than conventional braces. Patients adapt faster and experience improved functional comfort. Future research should focus on biomechanical refinements and long-term neuromuscular adaptation.

## Figures and Tables

**Table 1:** Comparison of speech parameters between groups

**Parameter**	**Clear Aligners (Mean ± SD)**	**Conventional Braces (Mean ± SD)**	**p Value**
Speech intelligibility (%)	92.8 ± 3.1	86.4 ± 4.2	<0.001 *
Articulation errors (/s/,/ʃ/,/t/,/d/) (n per 50 words)	1.2 ± 0.8	3.5 ± 1.1	<0.001 *
VAS speech difficulty (0-10)	2.3 ± 1.5	4.8 ± 1.9	<0.01 *
*Significant at p < 0.05

**Table 2:** Comparison of mastication parameters between groups

**Parameter**	**Clear Aligners (Mean ± SD)**	**Conventional Braces (Mean ± SD)**	**p Value**
Chewing cycles to color change (n)	18.4 ± 2.3	23.1 ± 2.9	<0.001 *
Masticatory efficiency (%)	82.5 ± 5.8	73.2 ± 6.1	<0.01 *
VAS mastication difficulty (0-10)	2.6 ± 1.4	5.1 ± 2.0	<0.001 *
*Significant at p < 0.05
